# Design Strategy for the EPR Tumor-Targeting of 1,2-Bis(sulfonyl)-1-alkylhydrazines

**DOI:** 10.3390/molecules26020259

**Published:** 2021-01-06

**Authors:** Philip G. Penketh, Hugh S. Williamson, Raymond P. Baumann, Krishnamurthy Shyam

**Affiliations:** 1 Department of Pharmacology, Yale University School of Medicine, New Haven, CT 06510, USA; baumannray@gmail.com (R.P.B.); krishnamurthy.shyam@gmail.com (K.S.); 2 Independent Mathematical Consultant, Middlesex TW16 7LN, UK; hugh.s.williamson@gmail.com

**Keywords:** anticancer drugs, targeting, EPR, activity confinement, sulfonylhydrazine, Evans blue, serum albumin, diffusion, half-life

## Abstract

A design strategy for macromolecular prodrugs is described, that are expected to exhibit robust activity against most solid tumor types while resulting in minimal toxicities to normal tissues. This approach exploits the enhanced permeability, and retention (EPR) effect, and utilizes carefully engineered rate constants to selectively target tumor tissue with short-lived cytotoxic moieties. EPR based tumor accumulation (half-life ~ 15 h) is dependent upon the ubiquitous abnormal solid tumor capillary morphology and is expected to be independent of individual tumor cell genetic variability that leads to resistance to molecularly targeted agents. The macromolecular sulfonylhydrazine-based prodrugs hydrolyze spontaneously with long half-life values (~10 h to >300 h dependent upon their structure) resulting in the majority of the 1,2-bis(sulfonyl)-1-alkylhydrazines (BSHs) cytotoxic warhead being released only after tumor sequestration. The very short half-life (seconds) of the finally liberated BSHs localizes the cytotoxic stress to the tumor target site by allowing insufficient time for escape. Thus, short lifespan anticancer species are liberated, and exhibit their activity largely within the tumor target. The abnormal tumor cell membrane pH gradients favor the uptake of BSHs compared to that of normal cells, further enhancing their selectivity. The reliance on physicochemical/chemical kinetic parameters and the EPR effect is expected to reduce response variability, and the acquisition of resistance.

## 1. Introduction

The concept for this chemotherapeutic project originated from researchers in the laboratory of the late Professor Alan C. Sartorelli in the Yale Medical School’s Department of Pharmacology. Following Professor Sartorelli’s death, his laboratory was closed, orphaning this promising project.

**Figure 1:**
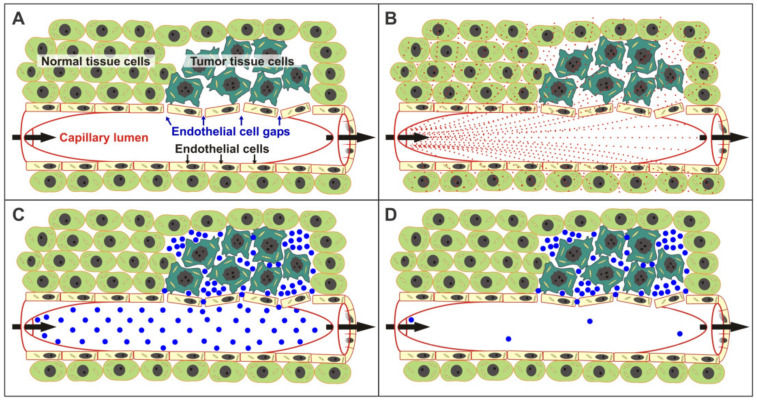
Illustration of the enhanced permeability and retention (EPR) effect, a verified phenomenon exclusive to solid tumors (**A**) Solid tumors, unlike normal tissues, have large gaps between the capillary endothelial cells permitting large scale extravasation and retention of macromolecules. (**B**) Small permeable molecules represented by small red dots readily traverse the endothelial cells lining capillary walls and penetrate both normal and tumor tissues. (**C**) Large membrane impermeable macromolecular prodrug (MW ~ 70 kDa) essentially confined to the plasma volume except in tumor tissues where the abnormally leaky vascular networks result in large molecules concentrating within the extracellular space. (**D**) Macromolecular prodrug molecules are retained at high levels in the extracellular tumor space long after concentrations within the plasma have diminished.

The EPR effect is a verified phenomenon exclusive to solid tumors that has great potential for therapeutic exploitation. The EPR effect arises from the unique architecture of solid tumor capillary networks. Solid tumors, unlike normal tissues, have large gaps between the capillary endothelial cells permitting large scale extravasation and retention of macromolecules (Figure 1), functioning in an analogous manner to a molecular fish trap. EPR effect-based targeting strategies are therefore not influenced by individual tumor cell genetic variability that may confer resistance to molecularly targeted agents. Treatment strategies that efficiently exploit the EPR effect would be of very wide utility, being applicable to most classes of solid tumors. For this approach to function, the cytotoxic activity, once delivered, must remain confined to the tumor. The very short tunable half-lives of the 1,2-bis(sulfonyl)-1-alkylhydrazines (BSHs) accomplish this function.

The EPR effect has successfully served as a means of improving the tumor selectivity of some clinically used antitumor agents by incorporating them into nanoparticle/macromolecule tumor delivery platforms [1,2,3]. However, the use of approved drugs is likely an extremely poor choice for maximizing tumor selectivity in most cases. This is because their success as ‘free-agents’ is due in part to pharmacokinetic properties favoring good distribution and tissue penetration, and these very same properties will also aid in the escape and redistribution of a formerly targeted agent. Thus, leakage and redistribution of tumor-targeted material to normal tissues is a major, and commonly occurring problem, with targeted drugs. Other major concerns include: the inefficient release of the targeted agent, inappropriate systemic activation, high sensitivity of normal tissues to the targeted agent, and non-benign delivery platform residual products.

1,2-Bis(sulfonyl)-1-alkylhydrazines (BSHs), developed in the Sartorelli laboratory, have a library of properties that allow them to largely circumvent these problems giving them significant promise as therapeutic warheads for EPR targeting platforms [4]. Despite these significant BSH advantages, EPR effect-based tumor-targeting strategies have yet to be explored with this class of agents.

Evans Blue (EB) is a low-toxicity, colorimetrically quantifiable azo dye with an extremely high binding affinity for serum albumin. EB is often used to estimate the proportion of body water contained in plasma since the EB/serum albumin complex does not significantly penetrate normal tissues or cells. The tightly bound EB/serum albumin complex behavesin vivo like a single macromolecular agent (mw ~ 70 kDa) selectively concentrating within solid tumors with their abnormally leaky vascular networks. One day after i.v. injection, the EB serum albumin complex is largely lost from the plasma and confined to solid tumor sites, where it persists for weeks, [1,2,3].

During our development of BSH prodrugs a number of latentiating linkers were identified which spontaneously fragmented with long half-lives under biological conditions ranging from ~10 h to >300 h. Covalent BSH prodrugs utilizing such linkages to either EB/analogs thereof, or to serum albumin directly, would be expected to release the vast majority of their short-lived cytotoxic payload only after these macromolecular complexes were confined to the tumor intracellular spaces, since this half-life is much greater than the half-life for EPR sequestration (~15 h) of the macromolecular prodrug. The small proportion of BSH warhead released within the bulk circulation would be relatively innocuous; and this minor fraction would not be expected to exceed normal tissue toxicity thresholds.

The EPR effect arises because solid tumors have abnormally large gaps between their capillary endothelial cells that permit large scale extravasation and retention of macromolecules (Figure 1) [1]. The EPR effect is not seen in normal healthy tissues. Since EPR targeting is primarily dependent upon abnormal tumor capillary morphology, it is not greatly influenced by individual tumor cell genetic variability that might lead to resistance to molecularly targeted agents. The preferential delivery and localization of cytotoxic stress to the tumor is assured by the interplay of several kinetic processes. The reliance on a combination of physicochemical/chemical kinetic parameters and the EPR effect is expected to reduce response variability and the acquisition of resistance compared to therapies which require direct activation of agents by variable levels of enzymes in tumor/normal tissues and/or the presence of differentially expressed molecular markers/targets. In the proposed strategy the tumor-selective delivery of cytotoxic stress is based upon three kinetic parameters that occur on different timescales. The first concerns the kinetics of the transfer of a macromolecular serum albumin prodrug complex from the plasma into tumor tissues. In the case of Evans Blue (EB)/serum albumin complexes, these occur with T_1/2_ ~ values of ~5 h and ~15 h, respectively [1,2,3]. These complexes are then retained in tumor tissues at near constant levels for hundreds of hours [1,2,3]. The second kinetic parameter involves the slow spontaneous hydrolysis/cleavage of a latentiating linker (T_1/2_ ~ 10 h to >300 h depending upon structure). This ensures that the vast majority of the fragmentation to release the 1,2-bis(sulfonyl)-1-alkylhydrazine (BSH) warhead occurs only after the bulk of the macromolecular prodrug has left the plasma and resides within the tumor delivery site. The third kinetic parameter is the rapid delivery of cytotoxic stress resulting from the secondary fragmentation of the liberated highly permeable BSH to generate cytotoxic electrophiles. This latter reaction occurs with precisely tunable T_1/2_ values (dependent upon the BSH structure) of between 1s to 280s [4,5,6]. The rapidity of this reaction limits the diffusion range of the BSH and safeguards against the dilution of the cytotoxic stress by escape from the tumor environment, and protects normal surrounding tissues from cytotoxic exposure [4]. In the detailed kinetic modeling study of targeted BSH cytotoxic stress delivery, it was determined that a half-life of approximately 8s would be optimal [4]. This allows sufficient time for the agent to act on cells proximal to the delivery site, but insufficient time for a significant proportion of the agent to enter the capillaries and be lost from the target site. Differences in the magnitude and direction of trans plasma membrane pH gradients between normal and cancer cells [7,8] will further decrease the relative toxicity of the weakly acidic BSHs to normal cells, while maximizing the anticancer activity of the BSH released within the tumor extracellular environment.

Targeting strategies based upon the EPR effect have been applied to existing therapeutics to endow them with greater tumor selectivity with some clinical success [1]. These delivery systems have been shown to “safely” transport payloads of cytotoxic molecules to tumors while largely shielding normal cells from their toxic effects during the transport process [1]. However, inefficient release, inappropriate systemic release, ineffective tumor uptake, and the leakage of the targeted cytotoxic material from the tumor site have remained major problems [9]. The temptation to press-gang existing clinical agents with proven anticancer activity into the role of “therapeutic warhead” for tumor-targeted systems should thus be explicitly avoided, because their success as “free-agents” is due, at least in part, to pharmacokinetic properties favoring good distribution and tissue penetration. However, these properties aid in the escape and systemic distribution of the formerly targeted agent. BSHs have properties that circumvent many of these failings and set them apart from other “therapeutic warheads” [6]. BSHs can be released by simple spontaneous chemical activation mechanisms that are independent of the presence, or differences in the levels of, activating enzymes or tumor specific molecular markers. This removes biological variables influencing the degree of targeting and/or tumor response [6,10,11]. *O*^6^-methylguanine-DNA methyltransferase (MGMT), the protein primarily responsible for BSH resistance, can provide a strong yet limited “toxicity threshold/buffer”, allowing the tolerance of BSH leakage from the target site and undesired background systemic activation [12]. However, the limited MGMT protection threshold can be overwhelmed within the tumor with targeted BSH delivery [13]. Moreover, MGMT is likely amenable to tumor-selective modulation in solid tumors increasing their preferential sensitivity towards chloroethylating BSHs [6,14], and the presence of MGMT deficient tumors in 5–20% of patients (dependent upon the tumor type) may permit the pre-selection of patients likely to exhibit exceptional responses [15,16,17].

Despite the possession of this combination of desirable properties, BSHs have yet to be utilized as therapeutic ‘warheads’ for EPR effect-based tumor targeted drug delivery platforms.

Several simple low molecular weight BSH prodrugs have shown remarkable in vivo activity, the most successful being laromustine (a.k.a. cloretazine, onrigin, VNP40101M, 101M) [6]. Laromustine is a spontaneously activating prodrug of the chloroethylating BSH 90CE [11]. Laromustine liberates 90CE in a mildly tumor selective manner based upon pH dependent activation and uptake [18,19].This indicates the therapeutic potential of BSHs even without the benefits of EPR dependent tumor-targeted delivery.

**Figure 2:**
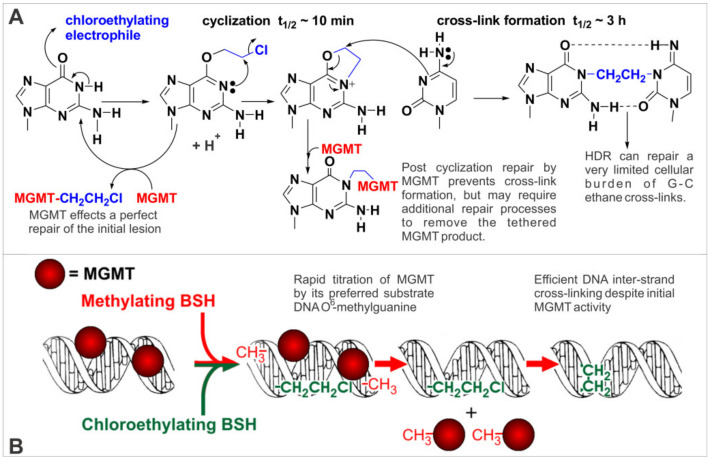
Chloroethylation of the *O*-6 position of DNA guanine, its sequelae, and the synergistic cytotoxicity of guanine *O*-6 methylating and chloroethylating BSHs. (**A**) Chloroethylation of guanine *O*-6 results in the formation of *O*^6^-(2-chloroethyl)guanine which rapidly rearranges to form the N1,*O*^6^-ethanoguanine cross-link precursor; this lesion then slowly transitions into highly cytotoxic G-C ethane cross-links. Points of repair/cross-link precursor quenching by MGMT are indicated. A limited number of G-C ethane cross-links can be repaired via HDR. Tumor selectivity arises predominantly from tumor deficits in one or more of these repair processes, with MGMT insufficiency likely being the foremost factor in most cases. (**B**) The synergistic cytotoxicity of guanine *O*-6 methylating and chloroethylating agents arises from the rapid titration of protective MGMT by the relatively low toxicity *O*^6^-methylguanine lesion, leaving an unimpaired path for the *O*^6^-(2-chloroethyl)guanine lesion to progress to highly cytotoxic G-C ethane cross-links.

BSHs react to generate greater yields of electrophiles that favor the alkylation of the *O*-6 position of DNA guanine, and more importantly, lower yields of other cytotoxic DNA reactive electrophiles with low tumor-selective cytotoxicity [6,19]. The cytotoxicity of BSHs exhibits the greatest MGMT activity dependency known [12,20,21,22,23,24]. In the case of 90CE, MGMT expression can result in a ~20-fold increase in the IC_50_ value, compared to only a 2 to 3-fold difference for bis-chloroethylnitrosourea (BCNU) in the same matching cell lines [12,24]. An MGMT molecule can only repair a single guanine *O*-6 lesion [23]. Once a cell’s MGMT reserves are titrated, repair ceases until fresh MGMT is synthesized [25]. This results in a temporal window of high sensitivity to guanine *O*-6 alkylation. In the case of the methylating BSH, 1,2-bis(methylsulfonyl)-1-(methyl)hydrazine (KS90), cytotoxicity is not manifested until all the cellular MGMT is titrated [12]. Unfortunately, guanine *O*-6 methylations are of a relatively low cytotoxicity even in the absence of repair [12]. However, KS90 is a relatively efficient MGMT titrator, resulting in 5–6 times the molar yield of guanine *O*-6 alkylations than its chloroethylating counterpart 90CE [12]. Thus, EPR targeted KS90 could be used to deplete tumor MGMT and selectively sensitize tumor cells to the more cytotoxic *O*^6^-(2-chloroethyl)guanine assault, thus resulting in synergistic tumor cell kill (Figure 2).

Guanine *O*-6 chloroethylators behave differently from methylators since the initial lesionspontaneously transitions into a highly cytotoxic1-(*N*^3^-cytosinyl),-2-(*N*^1^-guaninyl)ethane DNA-DNA interstrand cross-link (G-C ethane cross-link) via a *N*^1^,*O*^6^-ethanoguanine intermediate [26,27,28] (Figure 2). Cells have a small limited capacity to repair G-C ethane cross-links (an MGMT independent process) [26]. Thus, for a cell to survive, the MGMT activity must be sufficiently large to produce a repair rate that clears the guanine *O*-6 cross-link precursor lesions before a small but lethal number have transitioned into G-C ethane cross-links [26].A large excess of MGMT relative to the number of *O*-6 guanine lesion number is therefore required for resistance, but little MGMT depletion occurs even at highly cytotoxic doses [12,26]. Thus, MGMT expression results in strong resistance to both methylating and chloroethylating BSHs. The protection afforded by MGMT towards 90CE has additional limitations, since it is only proportional to the MGMT activity up to ~10,000 MGMT molecules/cell [12]. Beyond this point very large increases in MGMT expression result in only incremental additional resistance. Thus with efficient targeting, 90CE can kill cells even with exceptional MGMT levels, yet normal cellular MGMT levels can still provide a robust cytotoxicity threshold to cope with a significant degree of leakage/errant release [13]. A cytotoxicity threshold is an important parameter to achieve highly selective cancer cell kill with targeted agents due to inherent inaccuracies present in most targeting platforms. A targeted ricin-like molecule for example, where a single molecule can kill a cell [29] (ultimately potent with no threshold before toxicity is observed), can only tolerate a very small amount of errant release before widespread toxicity to normal tissues occurs.

**Spontaneously Hydrolyzing BSH “Warhead” Linkers.** A number of classes of BSH prodrugs have been previously synthesized by substitution of the *N*-2 proton with groups that are cleaved either spontaneously, or by particular enzyme classes under specific conditions [10,11,29,30,31,32,33]. Of particular interest to this application are a series of 2-aminocarbonyl-BSH derivatives in which both available positions on the amino group have been substituted. Prodrugs utilizing these linkages spontaneously liberate active BSH by hydrolyzing very slowly under normal biological conditions (pH 7.4 and 37 °C) with half-lives ranging from ~10 h to >300 h. BSHs utilizing two other linkage types (acyl and carbamate) also resulted in some compounds with suitably slow spontaneous cleavage rates in aqueous solutions. It is expected that the measured spontaneous hydrolysis rates of these moieties in these simple model compounds will be comparable to the rates that will occur in more complex structures. The spontaneous cleavage/hydrolysis of some of these masking groups (carbamates in particular) may at least in part be acid catalyzed. A long half-life for the release of the cytotoxic warhead from the macromolecular prodrug outside of the tumor environment is essential to give time for tumor accumulation and to minimize the release of BSH in the general circulation, but once sequestered within the tumor the activation rate is less important. Since serum albumin/EB complexes do not penetrate most normal tissues or cells to any large extent, and are normally confined to the plasma volume, [1,2,3] the serum albumin/EB-BSH and serum albumin-BSH prodrugs should be similarly restricted. This extracellular plasma confinement greatly restricts primary metabolism due to the very limited number of active enzymes in the plasma capable of acting upon them. However, it is possible that some types of plasma esterases etc. could have some limited activity on some of the proposed BSH linkers. The reliance on slow spontaneous non-enzymatic hydrolysis to liberate the active BSH under biological conditions should help minimize variability between animals and result in the bulk of the macromolecular prodrug becoming tumor associated in its intact form. Three suitable ‘linker’ types utilized in previous non-macromolecular BSH prodrugs designs [11,32,33] have appropriate spontaneous cleavage rates. Moreover, these linkages do not pose any synthetic challenges.

**Figure 3:**
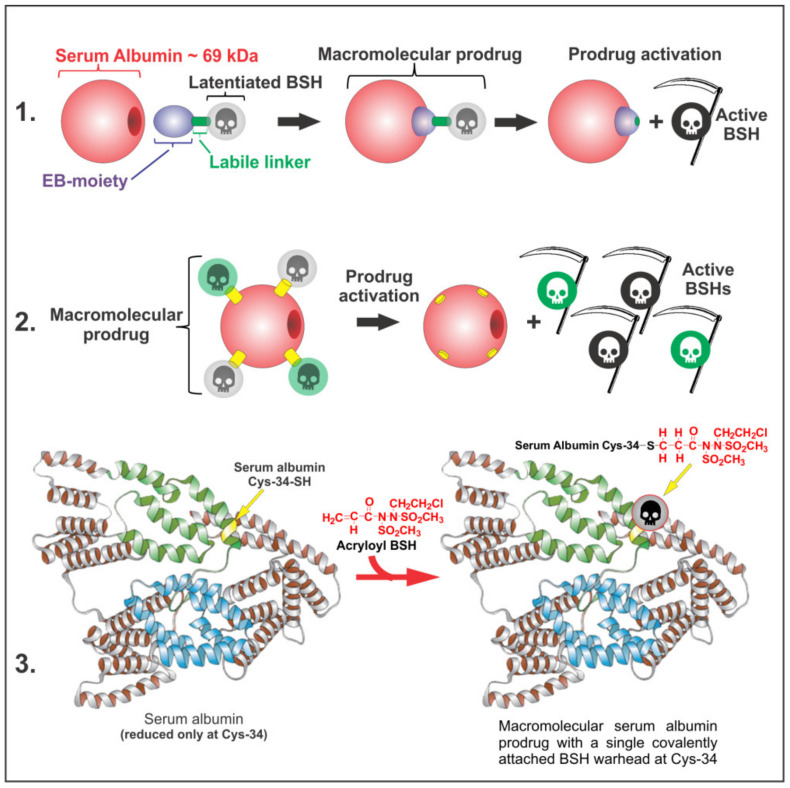
Proposed designs for EPR targeted macromolecular BSH prodrugs. (**1**). Design utilizing a BSH coupled via a slowly hydrolyzing linker (T_1/2_ ~ 10 to >300 h) to an EB analog, the tight non-covalent serum albumin binding generates a macromolecular prodrug. (**2**). Alternative design utilizing BSHs coupled directly to serum albumin via available cysteine thiols. This design enables the coupling of multiple and dissimilar/synergistic BSH molecules. (**3**). The direct covalent BSH warhead coupling to serum albumin showing the location of Cys-34. A macromolecular prodrug can be generated containing a single BSH warhead at Cys-34 (or multiple BSH warheads depending upon the level of serum albumin reduction) by the Michael addition of acryloyl-BSH to serum albumin thiol groups.

**Serum Albumin Attachment Strategies.** Two serum albumin attachment strategies are proposed, (Figure 3) the first involves the covalent attachment of a BSH to an EB analog via a slowly hydrolyzing linker. The synthetic chemistry is based on methods recently used to synthesize EB analogs designed as tumor imaging agents [34]. The direct attachment to EB analogs has several advantages; (i) the well-established tumor-selective localization of EB/analogs thereof; (ii) EB analogs are highly chromophoric permitting facile quantification and simplifying tissue/tumor distribution studies; (iii) the resultant EB-BSH covalent complexes are relatively small molecules (~1250–1500 kDa) that generate the macromolecular agent in vivo upon tight non-covalent binding to serum albumin. The direct covalent attachment of BSHs to serum albumin (Figure 3), has the following advantages: (i) the ability to readily attach BSH + linker moieties to serum albumin thiol groups via a simple one step Michael type addition reaction; (ii) the potential ability to attach multiple BSH molecules per serum albumin molecule due to the large number of cysteine SH groups present in heavily reduced preparations; (iii) the ability to attach multiple synergistic BSH types in a favored ratio (e.g., a synergistic combination of methylating and chloroethylating BSHs), (Figure 3); (iv) high aqueous solubility. Serum albumin contains 35 cysteine residues, but normally only one presents a free thiol group (Cys34) as the remaining 34 are involved in 17 disulfide bonds [35]. Typically serum albumin preparations contain around 0.6 moles of free Cys34 thiol per mole of serum albumin with the remaining 0.4 moles of Cys34 being involved in disulfide bonds with low molecular weight thiols (cysteine, GSH etc.) [35]. If these serum albumin preparations are reduced with dithiothreitol (DTT) under mild conditions then separated, samples which are essentially fully reduced at only Cys34 are readily produced [36]. The reduced serum albumin is then easily separated from these low molecular weight components by ultrafiltration. Longer reductions under harsher, more denaturing conditions result in progressively more –*S*-*S*- reduction [37], the time course and the number of thiol groups generated per mole of serum albumin being easily determined in the isolated protein using Ellman’s reagent. Serum albumin-based prodrugs containing a single BSH linked to Cys34 should be the easiest to prepare; furthermore, this is expected to minimally affect the behavior of this protein and generate a uniform product (Figure 3). However, serum albumin BSH prodrugs with up to 10 covalently attached BSH molecules could be synthesized and evaluated for BSH release. The attachment of acryloyl-BSH warheads to serum albumin could be carried out by reacting a slight molar excess of acryloyl-BSH per reduced thiol residue in the serum albumin preparation in phosphate buffer saline. A 5 min reaction at 37 °C, followed by washing/ultrafiltration/lyophilization should yield the desired macromolecular BSH prodrug.

## 2. Results

**Figure 4:**
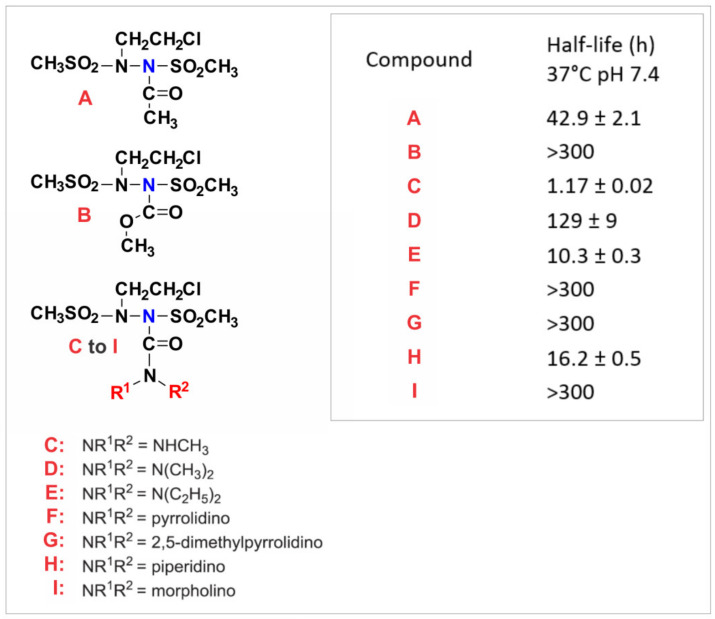
The half-life of simple 90CE prodrugs with different N-2 substituents. The half-life/spontaneous hydrolysis rate of various low molecular weight 90CE prodrugs was determined (37 °C, pH of 7.4). It was noted that the stability of some of these linkages would allow sufficient time for macromolecular prodrug analogs to be accumulated by tumor tissues before any significant systemic activation occurred.

**Linker half-life determination.** The hydrolytic half-lives of several proposed linker moieties were determined using the proton release assay. The results are given in (Figure 4), several possible linkers with suitably long half-live values (40–300 h) were identified.

**Synthesis and Evaluation of Macromolecular Prodrugs.** Due to the closure of the laboratory, the final agents were never produced and evaluated in any animal models. However, these molecules are anticipated to be relatively easy to synthesize, even in modestly equipped chemical synthesis laboratories, express broad anticancer activity towards solid tumors, and more efficiently exploit the EPR effect than previous attempts. Thus this represents a very promising line of research for anticancer drug development groups, even with limited equipment and funding.

### 2.1. Mathematical Analysis

**Figure 5:**
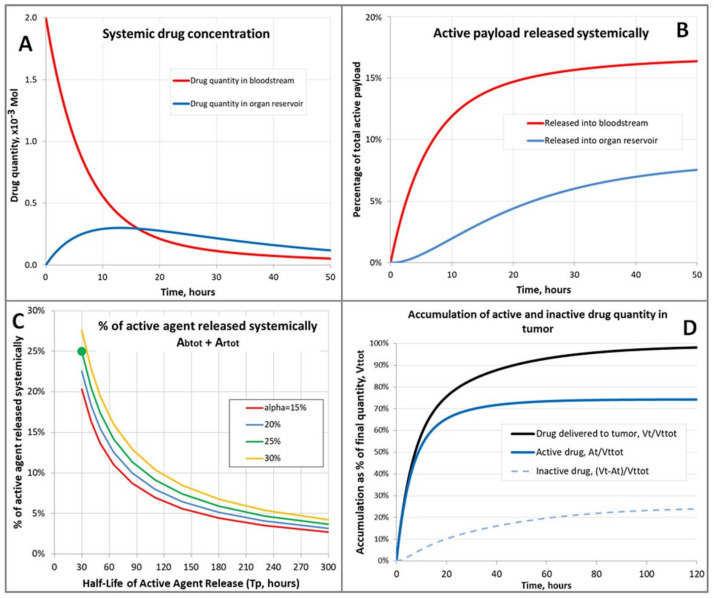
Model of drug release and active agent accumulation. Input parameters (*T_b_*, *T_r_*, *T_p_*, *V*) are per the example in the text. (**A**) Systemic drug concentration; (**B**) Active payload released sys-temically; (**C**) % of active released systemically; (**D**) Accumulation of active and inactive drug quantity in tumor. In panel (**C**), *T_p_* varies–the green dot shows the example given in the text.

**Overview and assumptions.** If you examine the experimental data [2,3], you will immediately notice that the curves representing the bloodstream and tumor levels of the macromolecular prodrug do not match the simplistic images shown in (Figure 1). If they did, the experimental data would show matching relative changes in the concentration of the macromolecular prodrug in the bloodstream and tumor tissue. That is when half the material was lost from the bloodstream, half the material would have accumulated in the tumor etc. Thus, they would have equivalent half times, although the peak absolute concentrations would not be the same as this would be determined by the relative volumes of these two compartments. If we look at the decrease in the levels of macromolecular prodrug in the bloodstream we notice that it initially follows first order kinetics (half-life, ~5 h), but then markedly deviates from this towards the end showing a very protracted final decrease.In the tumor tissue the macromolecular prodrug accumulates with a very different half-life value of ~15 h. These two observations are not possible with just the two compartments illustrated in (Figure 1). If we include a third compartment of muscle, liver, and kidney as shown in the experimental data [2,3], which acts as a reservoir for the agent, and a small but limited excretion/metabolism of the macromolecular prodrug, the experimental data can be very accurately matched. In our model we have assumed a ~35 g mouse has a total blood volume of ~1.7 mL, a liver mass 1.5 g, kidneys of total mass of 0.5 g, and 10 g of muscle mass. Thus, the size of the liver, kidney, muscle reservoir is 12 g. Assuming the experimental tumors have a mass of ~0.02 g (volume ~20 µL) we can very accurately model the experimental data shown in [2,3]. For a macromolecular prodrug with a linker with a hydrolytic half-life (Tp) of ~30 h, it calculated that ~16% of the drug’s active “payload” is released into the bloodstream, ~7.5% into the reservoir of liver/kidney/muscle, and ~80% of the drug that enters the small tumor compartment still has its cytotoxic payload. This represents extremely good tumor-selective targeting, especially considering that macromolecular prodrugs with a longer linker hydrolytic linker half-life value would perform better (Figure 5C,D). Furthermore, the BSH cytotoxicity threshold should largely spare most normal tissues from their relatively small cytotoxin exposure. This mathematical model suggests that the dose of the macromolecular prodrugrequired should be dependent upon the tumor burden, rather than the mass of the recipient patient, which is what one would intuitively expect for a truly tumor selective agent. This would result in a lower required dose of the macromolecular prodrug, with concomitantly lower toxicity to the patient, if the treatment was initiated earlier in the course of the disease when the tumor burden was low.

### 2.2. Mathematical Analysis of Drug Delivery

A mathematical model incorporating bloodstream, organ reservoir and tumor was developed in an attempt to reproduce the observed pharmacodynamics [2,3], and to predict key measures of the likely efficacy of this drug delivery mechanism. Surprisingly (and gratifyingly) the model yielded analytical solutions for these key measures, allowing their dependency on the input assumptions to be calculated directly, rather than using numerical methods.

There follows a description of the model used and the analytical solutions it yields. The derivation of the solution is straightforward but somewhat protracted, so only a summary is presented here.

**The Model**

- A quantity (*V*) of drug is introduced to the bloodstream at time zero.
- The drug contains an active ‘payload’—targeted at a tumor—which releases in the body with half-life $\left(T_{p}\right)$
- The drug exits the bloodstream with half-life $\left(T_{b}\right)$.
- A constant fraction (α) of the drug exiting the bloodstream enters a “reservoir” of organs. The reservoir returns the drug back to the bloodstream with half-life $\;(T_{r})$.
- The drug enters the tumor at a rate proportional to its concentration in the bloodstream and is retained.
- The drug exiting the bloodstream which neither enters the reservoir nor the tumor is excreted or otherwise lost to the system.

**Required Model Outputs**

It is required to find:

- The quantity of drug which releases its active payload into the bloodstream, $A_{btot}$
- The quantity of drug which releases its active payload into the reservoir, $A_{rtot}$
- Of the drug which enters the tumor, the fraction *Q* which retains its active payload

**Solution**

(1) $$A_{btot}=\frac{\beta \left(1+\rho \right)V}{\left(1+\beta \right)\left(1+\rho \right)-\alpha }$$

(2) $$A_{rtot}=\frac{\alpha \rho V}{\left(1+\beta \right)\left(1+\rho \right)-\alpha }$$

(3) $$Q=\frac{\left(1-\alpha \right)\left(1+\rho \right)}{\left(1+\beta \right)\left(1+\rho \right)-\alpha }$$

where $\beta =T_{b}/T_{p}$ and $\rho =T_{r}/T_{p}$.

As might be expected, the results depend only on the ratios between the system half-lives and not their absolute values.

**An Example**

- Half-life of drug in bloodstream = $T_{b}$ = 5 h
- Half-life of drug in reservoir of organs = $T_{r}$ = 15 h
- Half-life of active payload within drug = $T_{p}$ = 30 h
- Quantity of drug= *V* = 2.0 × 10^−3^ Mol
- Proportion of drug exiting bloodstream which enters reservoir of organs = α = 25%
- β = 0.1667;  ρ = 0.5
- $A_{btot}$= $\frac{0.002\;\times 0.25}{1.5}$= 3.33 × 10^−4^ Mol
- $A_{\mathrm{rtot}}$= $\frac{0.002\;\times 0.125}{1.5}$= 1.67 × 10^−4^ Mol
- Q = $\frac{1.125}{1.5}$ = 75%

Figure 5 uses this example to illustrate the dynamics predicted by the model.

**Summary of Solution Derivation**

*Drug Quantities in Bloodstream and Reservoir*

Define the following:

$V_{b}\left(t\right)$,$V_{r}\left(t\right)$ = quantity of drug in bloodstream and reservoir respectively.

$K_{b}=\frac{log_{e}2}{T_{b}}$;$\;K_{r}=\frac{log_{e}2}{T_{r}}$.

These are the exponential decay constants for the drug in the bloodstream and the reservoir respectively.

The initial conditions are $V_{b}\left(0\right)=V$, $V_{r}\left(0\right)=0$.

The drug exits the bloodstream at a rate proportional to its concentration, and re-renters from the reservoir at a rate proportional to the concentration there:

(4) $$\frac{dV_{b}}{dt}=-K_{b}V_{b}+K_{r}V_{r}$$

Likewise, the drug exits the reservoir at a rate proportional to its concentration and enters from the bloodstream at a rate proportional to the concentration there:

(5) $$\frac{dV_{r}}{dt}=\alpha K_{b}V_{b}-K_{r}V_{r}$$

Standard calculus leads to the solution of these two simultaneous linear differential equations:

(6) $$V_{b}\left(t\right)=\frac{V}{x_{2}-x_{1}}\left\{\left(x_{2}+K_{b}\right)e^{x_{1}t}-\left(x_{1}+K_{b}\right)e^{x_{2}t}\right\}$$

(7) $$V_{r}\left(t\right)=\alpha K_{b}V\frac{e^{x_{2}t}-e^{x_{1}t}}{x_{2}-x_{1}}$$

where

(8) $$x_{1}=-\frac{K_{b}+K_{r}+\sqrt{{\left(K_{b}+K_{r}\right)}^{2}-4\left(1-\alpha \right)K_{b}K_{r}}}{2}$$

(9) $$x_{2}=-\frac{K_{b}+K_{r}-\sqrt{{\left(K_{b}+K_{r}\right)}^{2}-4\left(1-\alpha \right)K_{b}K_{r}}}{2}$$

*Payload Released to Bloodstream*

Define the following:

- $A_{b}\left(t\right)$ = the cumulative quantity of active payload released into the bloodstream by time *t*, so that $A_{b}\left(0\right)=0$.
- $K_{b}=\frac{log_{e}2}{T_{p}}$ = the exponential decay constant for the release of the active payload from the drug.

From the principles of exponential decay, a unit quantity of the drug will release its active payload at rate $K_{p}e^{-K_{p}t}$.

Thus the rate of release into the bloodstream is given by:

(10) $$\frac{dA_{b}}{dt}=V_{b}K_{p}e^{-K_{p}t}$$

Using the expression for $\;V_{b}\left(t\right)$ above, this expression may be integrated with respect to time to give:

(11) $$A_{btot}=\frac{K_{p}V}{x_{2}-x_{1}}\left\{\frac{x_{1}+K_{b}}{x_{2}-K_{p}}-\frac{x_{2}+K_{b}}{x_{1}-K_{p}}\right\}$$

which after some manipulation simplifies to:

(12) $$A_{btot}=\frac{K_{p}\left(K_{p}+K_{r}\right)V}{\left(K_{p}+K_{b}\right)\left(K_{p}+K_{r}\right)-\alpha K_{b}K_{r}}.$$

Substituting in half-life ratios defined by $\beta =K_{p}/K_{b}=T_{b}/T_{p}$ and $\rho =K_{p}/K_{r}=T_{r}/T_{p}$ gives the solution above.

*Payload Released to Reservoir of Organs*

Define $A_{r}\left(t\right)$ = the cumulative quantity of active payload released into the reservoir by time *t* so that $A_{r}\left(0\right)=0$.

The expression for the rate of release is exactly analogous to that for the bloodstream:

(13) $$\frac{dA_{r}}{dt}=V_{r}K_{p}e^{-K_{p}t}.$$

The same method of solution by integration and simplification leads to

(14) $$A_{rtot}=\frac{aK_{b}K_{p}V}{\left(K_{p}+K_{b}\right)\left(K_{p}+K_{r}\right)-\alpha K_{b}K_{r}}$$

and the solution above.

*Proportion of Active drug delivered to Tumor*

Define the following:

$V_{t}\left(t\right)$ = quantity of drug in tumor.

This is zero initially and increases at a rate proportional to the amount of drug in the bloodstream:

(15) $$V_{t}\left(0\right)=0$$

(16) $$\frac{dV_{t}}{dt}=qV_{b}$$

where *q* is a constant defining this rate.

Once again, we may integrate this expression with respect to time to yield:

(17) $$V_{ttot}=\frac{qV}{x_{2}-x_{1}}\left\{\frac{\left(x_{1}+K_{b}\right)}{x_{2}}-\frac{\left(x_{2}+K_{b}\right)}{x_{1}}\right\}$$

which may be simplified to:

(18) $$V_{ttot}=\frac{qK_{r}V}{\left(1-\alpha \right)K_{b}K_{r}}=\frac{qV}{\left(1-\alpha \right)K_{b}}$$

Define $A_{t}\left(t\right)$ = the cumulative volume of drug retaining its active payload to have entered the tumor by time *t*, so that $A_{t}\left(0\right)=0$.

The drug entering the tumor at time *t* retains a fraction $e^{-K_{p}t}$ of its active payload. Thus the tumor gains this active payload at rate:

(19) $$\frac{dA_{t}}{dt}=qV_{b}e^{-K_{p}t}$$

Once again integrating with respect to time and simplifying yields:

(20) $$A_{ttot}=\frac{q\left(K_{p}+K_{r}\right)V}{\left(K_{p}+K_{b}\right)\left(K_{p}+K_{r}\right)-\alpha K_{b}K_{r}}$$

The final proportion of drug with intact active payload delivered to the tumor is thus:

(21) $$Q=\frac{A_{ttot}}{V_{ttot}}=\frac{\left(1-\alpha \right)K_{b}\left(K_{p}+K_{r}\right)}{\left(K_{p}+K_{b}\right)\left(K_{p}+K_{r}\right)-\alpha K_{b}K_{r}}$$

Substituting in the half-life ratios as before gives the solution (3) above.

## 3. Conclusions

There exists strong experimental evidence to support the selective delivery and retention of macromolecular agents of a molecular mass of approximately 70 kDa by solid tumor tissues [1,2,3]. To effectively exploit the EPR effect as a basis for an anticancer therapy, the actions of the therapeutic agent must remain confined to the tumor tissue.This requires a therapeutic warhead(s) that is inactive until tumor delivery, but once the cytotoxin is activated, within the tumor tissue, it reacts extremely rapidly precluding systemic toxicities [4]. BSHs possess all the appropriate properties to fulfill this role, and offer significant promise as EPR targeted cytotoxins. Furthermore, EPR could be used to deliver BSH prodrug activating enzymes to tumors resulting in the liberation of short lived BSH within solid tumors though with reduced selectivity (4).

## 4. Materials and Methods

### Chemical Synthesis

No complex chemistry is envisioned for the synthesis of the described macromolecular BSH prodrugs, and a minimally equipped chemical synthesis laboratory should suffice.

**Figure 6:**
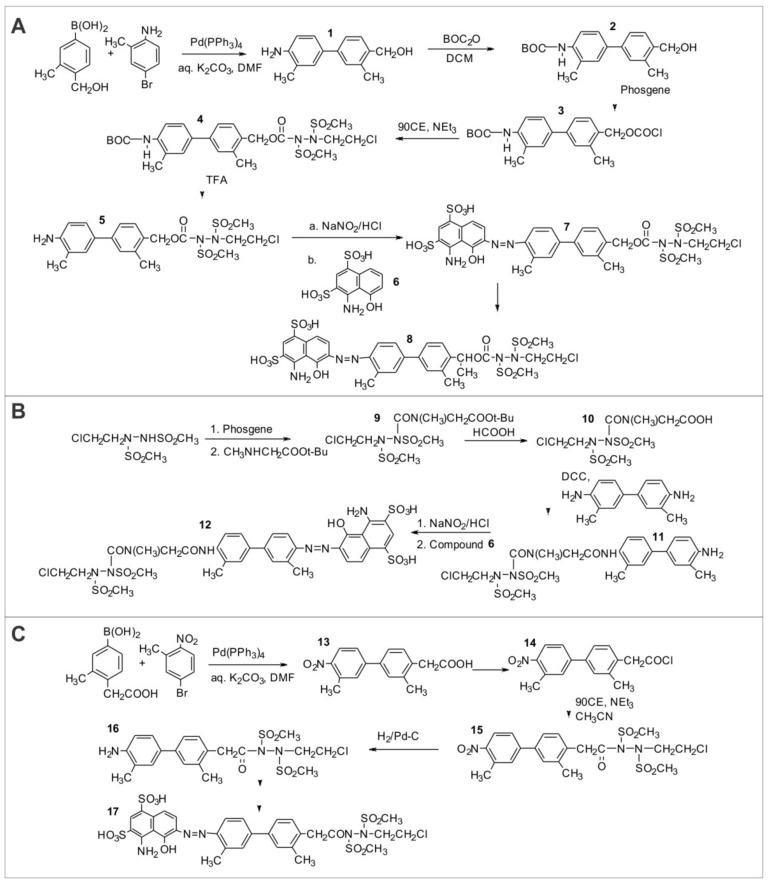
Chemical synthesis. (**A**) Synthesis of BSH-Evans Blue analog covalent conjugates involving carbamate linkages; (**B**) Synthesis of BSH-Evans Blue analog covalent conjugates involving aminocarbonyl linkages; (**C**) Synthesis of BSH-Evans Blue analog covalent conjugates involving acyl linkages.

**Synthesis of Evans Blue analogs with attached BSH warheads, and acryloyl BSH warheads.** Carbamates (**7** and **8**): 4-Amino-4′-(hydroxymethyl)biphenyl (**1**) could be prepared by a Suzuki cross-coupling reaction between 4-bromoaniline and 4-(hydroxymethyl)phenylboronic acid by heating the reactants under reflux in a mixture of 2M potassium carbonate and *N*,*N*-dimethylformamide in the presence oftetrakis(triphenylphosphine)palladium [38]. The t-BOC derivative of 1 (**2**), synthesized by reacting 1 with di-tert-butyl dicarbonate [39], are reacted with phosgene in the presence of pyridine in dichlormethane to give the chloroformate (**3**), which is then be reacted with 1,2-bis(methylsulfonyl)-1-(2-chloroethyl)hydrazine (90CE) to give the BOC-protected aminobiphenyl (**4**) [32]. Removal of the protective group by reaction with trifluoroacetic acid to give the free amine (**5**), followed by diazotization and coupling with the disulfonic acid (**6**) should give the target molecule (**7**) [34] (Figure 6A). A similar sequence of reactions could be used to synthesize the secondary carbamate (**8**) from the corresponding phenylboronic acid of the secondary alcohol, prepared from the commercially available 4-acetylphenylboronic acid.The carbamate linkage is expected to be more stable in (**8**) than in (**7**). Aminocarbonyl analog (**12**): The aminocarbonyl derivative (**12**) could be synthesized as shown.The tert-butyl ester (**9**) can be synthesized by reacting the chlorocarbonyl derivative of 90CE, formed by the reaction of triphosgene with 90CE in the presence of triethylamine in dioxane, with sarcosine tert-butyl ester. Acid-catalyzed deprotection of the tert-butyl group to give the free acid (**10**) [40], followed by condensation of (**10**) with 4,4′-diamino-3,3′-dimethylbiphenyl in the presence of dicyclohexylcarbodiimide (DCC) to yield (**11**) [39]. Diazotization of the free amino group, followed by the condensation of the diazonium ion with (**6**) should give the target molecule (**12**) [34] (Figure 6B). Acyl analog: 4-Carboxymethyl-4′-nitrobiphenl (**13**) could be synthesized by a cross-coupling reaction between 4-nitro-1-bromobenzene and 4-carboxymethylphenyl boronic acid [38]. Reaction of (**13**) with thionyl chloride should yield the acid chloride (14) [41], which can then be reacted with 90CE in the presence of triethylamine in acetonitrile to give 15 [33]. Reduction of (**15**) by catalytic hydrogenation to give (**16**), followed by diazotization and condensation as described earlier will give the acyl analog (**17**) [34] (Figure 6C) Acryloyl-BSHs could be synthesized in an analogous manner to the previously synthesized acyl-BSHs (**33**) but by using acryloyl chloride in place of an acyl chloride.

**Decomposition kinetics.** The kinetics of decomposition of various compounds and intermediates under various conditions can be followed using a spectrophotometric proton release assay. This is the preferred method for relatively short-lived agents as the decomposition can be followed in real time. This method is also suitable for following the decomposition of much longer-lived agents, as long as the reaction vessel/cuvette is sealed to prevent changes in pH due to CO_2_ absorption/desorption, and the temperature is closely controlled [5,11,18,42].

## Data Availability

All data is contained within the article.
